# Testing deep placement of an ^15^N tracer as a method for in situ deep root phenotyping of wheat, barley and ryegrass

**DOI:** 10.1186/s13007-019-0533-6

**Published:** 2019-12-09

**Authors:** Si Chen, Simon Fiil Svane, Kristian Thorup-Kristensen

**Affiliations:** 1 College of Agriculture and Biotechnology, Zhejiang University, Hangzhou, 310058 China; 2 Department of Plant and Environmental Science, University of Copenhagen, Højbakkegårds Alle, 13, 2630 Taastrup, Denmark

**Keywords:** ^15^N tracer, Deep rooting, Phenotyping, Drought stress gradients

## Abstract

**Background:**

Deep rooting is one of the most promising plant traits for improving crop yield under water-limited conditions. Most root phenotyping methods are designed for laboratory-grown plants, typically measuring very young plants not grown in soil and not allowing full development of the root system.

**Results:**

This study introduced the ^15^N tracer method to detect genotypic variations of deep rooting and N uptake, and to support the minirhizotron method. The method was tested in a new semifield phenotyping facility on two genotypes of winter wheat, seven genotypes of spring barley and four genotypes of ryegrass grown along a drought stress gradient in four individual experiments. The ^15^N labeled fertilizer was applied at increasing soil depths from 0.4 to 1.8 m or from 0.7 to 2.8 m through a subsurface tracer supply system, and sampling of aboveground biomass was conducted to measure the ^15^N uptake. The results confirm that the ^15^N labeling system could identify the approximate extension of the root system. The results of ^15^N labeling as well as root measurements made by minirhizotrons showed rather high variation. However, in the spring barley experiment, we did find correlations between root observations and ^15^N uptake from the deepest part of the root zone. The labeled crop rows mostly had significantly higher ^15^N enrichment than their neighbor rows.

**Conclusion:**

We concluded that the ^15^N tracer method is promising as a future method for deep root phenotyping because the method will be used for phenotyping for deep root function rather than deep root growth. With some modifications to the injection principle and sampling process to reduce measurement variability, we suggest that the ^15^N tracer method may be a useful tool for deep root phenotyping. The results demonstrated that the minirhizotrons observed roots of the tested rows rather than their neighboring rows.

## Introduction

Drought and nutrient deficiency are the main constraints limiting plant growth and productivity [1, 2]. It is a frequent occurrence for plants that water and nutrient uptake fail to meet requirements for growth. The grain yield is especially sensitive to resource deficiency during grain filling, when vegetative growth is limited and the absorption of water and nutrients mainly supports grain development [3–5]. To cope with this situation, crop species have evolved both structural and physiological traits to improve resource acquisition and utilization. Deep rooting is one of the most effective traits, which enable plants to access deep soil resources and support yield formation [6–9]. To improve drought tolerance and subsoil exploration, deep rooting is a desirable trait for plant breeders.

The expression of root traits and performance is a complex process under field conditions, which is regulated by the morpho-physiological abilities of the genotype, growth environment and interactions between them [10–12]. For example, roots can grow deeper to explore more resources when soil water and N become limiting in upper soil layers [13, 14]. Cereal roots mainly grow before flowering and the deep root traits form before reproductive growth [4–6]. Under terminal drought stress, deep rooting genotypes have the advantage of accessing extra water and nutrients for yield formation compared to shallow rooting genotypes. For example, an additional 10.5 mm subsoil water uptake from the 1.35–1.85 m soil layer after the flowering stage led to a yield increase of 0.62 t/ha in wheat [7]. The structure and functioning of roots vary among the genotypes and growth environment, which makes field root phenotyping a challenging task.

Considerable efforts have focused on the phenotyping of roots in soilless media, which provides visualization of the root system and efficient analysis of root images [15–17]. Such laboratory studies are conducted on young plants with roots grown in small containers under controlled environments, where the plants fail to reflect actual field root traits or root traits during the reproductive stage [18, 19]. These studies overlook the influences of soil hardiness, pore size and distribution, moisture and fertility on root traits in the field [20]. Therefore, direct field root phenotyping is essential.

The methods used to measure roots in the field can be categorized as (i) traditional excavation methods, such as trenching and soil coring [21, 22], (ii) nondestructive minirhizotrons, ground-penetrating radar and electrical capacitance [23–25], and (iii) indirect root activity estimations, e.g., tracers [26]. However, most of these methods are not suitable for deep root phenotyping because the methods do not efficiently detect root traits or functions for a large number of genotypes.

In general, there are few methods for root phenotyping available to plant breeders [21, 27]. Some studies have used soil coring and minirhizotron methods to identify deep rooting among a few genotypes [22, 28, 29]. The soil coring method involves the collection of a large number of soil samples and root washing, which are labor-intensive and time-consuming [30]. The soil coring method has been improved in the core-break method to enable rapid assessment of deep root distribution [22]. In contrast, a minirhizotron is a nondestructive method for field root phenotyping in situ, which provides a dynamic visualization of root growth [31, 32]. Nevertheless, a large investment is needed to establish a minirhizotron facility for field root phenotyping. Aiming at developing efficient and low-cost methods, the ^15^N tracer method is introduced in this study and is compared to the minirhizotron method for deep root phenotyping in situ. The ^15^N tracer method relies on the measurement of deep root uptake ability through deep placement of ^15^N-labeled nitrate [33, 34]. Additionally, a linear correlation of the ^15^N uptake and root density at different soil depths has been reported [26]. It is a potential phenotyping method for estimates of deep rooting traits and activity.

In this study, the ^15^N tracer method was tested in a new root phenotyping facility (RadiMax) to select deep rooting genotypes based on deep ^15^N uptake and to support the minirhizotron method by analyzing the neighboring effect. The ^15^N tracer uptake was further compared with the minirhizotron method for the identification of deep-rooted genotypes. The hypotheses of this study are that (1) deep ^15^N uptake will be correlated with the rooting depth of different crop species, (2) there will be genotypic differences in the deep ^15^N uptake related to deep root growth, and (3) deep roots of a crop row grow mainly below the crop row itself and do not use resources from their neighboring rows.

## Materials and methods

### Experimental design

**Fig. 1:**
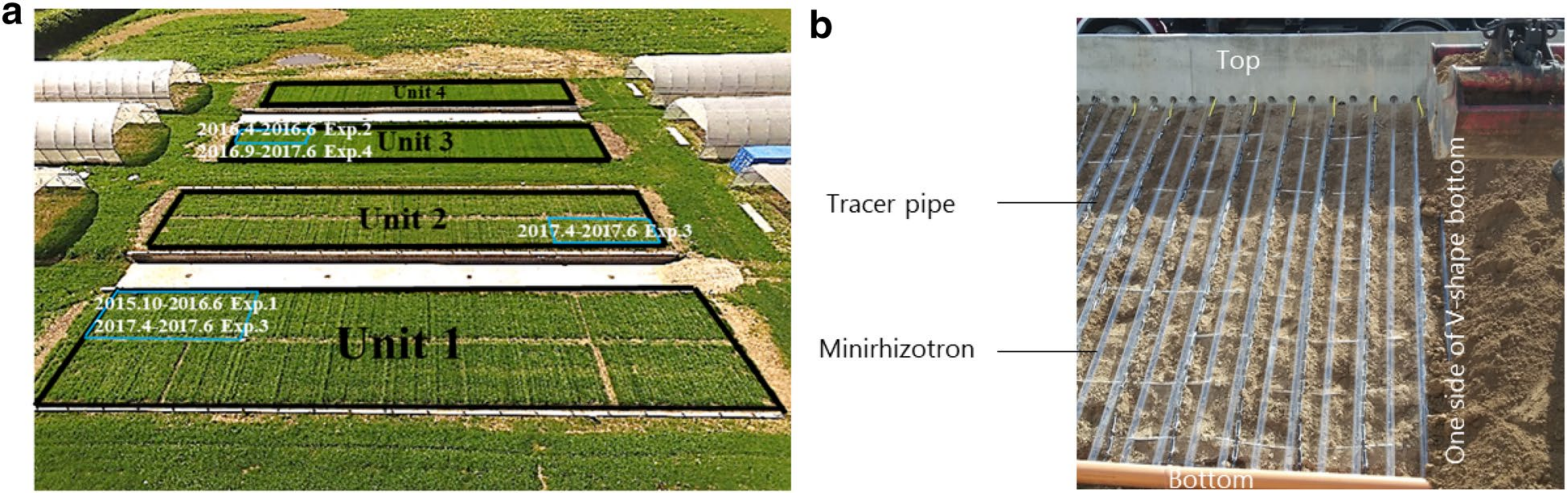
Experimental sites and methods used in the root phenotyping facility, RadiMax. **a** The four black squares show units 1, 2, 3 and 4 of the RadiMax facility. The blue squares in units 1, 2 and 3 show the experimental sites with 30 crop rows and the timelines of the four experiments. **b** The tracer pipes and minirhizotrons were set along the V-shaped bottom in each unit

Four experiments were carried out in a new root phenotyping facility, RadiMax (Fig. 1), which was built on a farm of the University of Copenhagen in Taastrup (55° 40́ 90.35ʹ N, 12° 18́ 24.84ʺ E), Denmark, in 2015. The ^15^N tracer method was applied at increasing depths along the crop rows to crops of winter wheat and spring barley to test deep ^15^N uptake and to estimate root depth. The first experiment (Exp. 1) was conducted on two genotypes of winter wheat, ‘Hystar’ and ‘Tabasco’, in 2016. The second experiment (Exp. 2) was conducted on two genotypes of spring barley, ‘Evelina’ and ‘Laurikka’, in 2016. The third experiment (Exp. 3) was conducted on seven genotypes of spring barley in 2017. Except for ‘Kenia’ and ‘Evelina’, others are modern genotypes. The fourth experiment (Exp. 4) was conducted on four genotypes of perennial ryegrass (*Lolium perenne* L.) in 2017, including diploid genotypes ‘Esquire’ and ‘Mercitwo’, and tetraploid genotypes ‘Fabian’ and ‘Tetrastar’ (Table 1).

**Table 1:** Outline of experimental agronomy and sampling events for four experiments in 2016 and 2017

Year	Exp	Unit	Species	Sowing day	^15^N application			Sampling
					(BBCH scale) Date	Volume of solution (L)	Amount of ^15^N (mg m^−1^)	
2016	1	1	Winter wheat	(00) 1 October 2015	(58) 2 June 2016	50.0	9.6	(89) 20 July 2016
	2	3	Spring barley	(00) 12 April 2016	(55) 10 June 2016	50.0	9.6	(89) 1 August 2016
2017	3	1	Spring barley	(00) 2 April 2017	(58) 16 June 2017	30.3	5.4	(69) 23 June 2017
		2				36.3	6.1	(89) 28 July 2017
	4	3	Ryegrass	(00) 27 September 2016	(39) 20 June 2017	43.5	6.7	(39) 15 July 2017

BBCH scale is shown in the parentheses. Exp. is an abbreviation for experiment

**Fig. 2:**
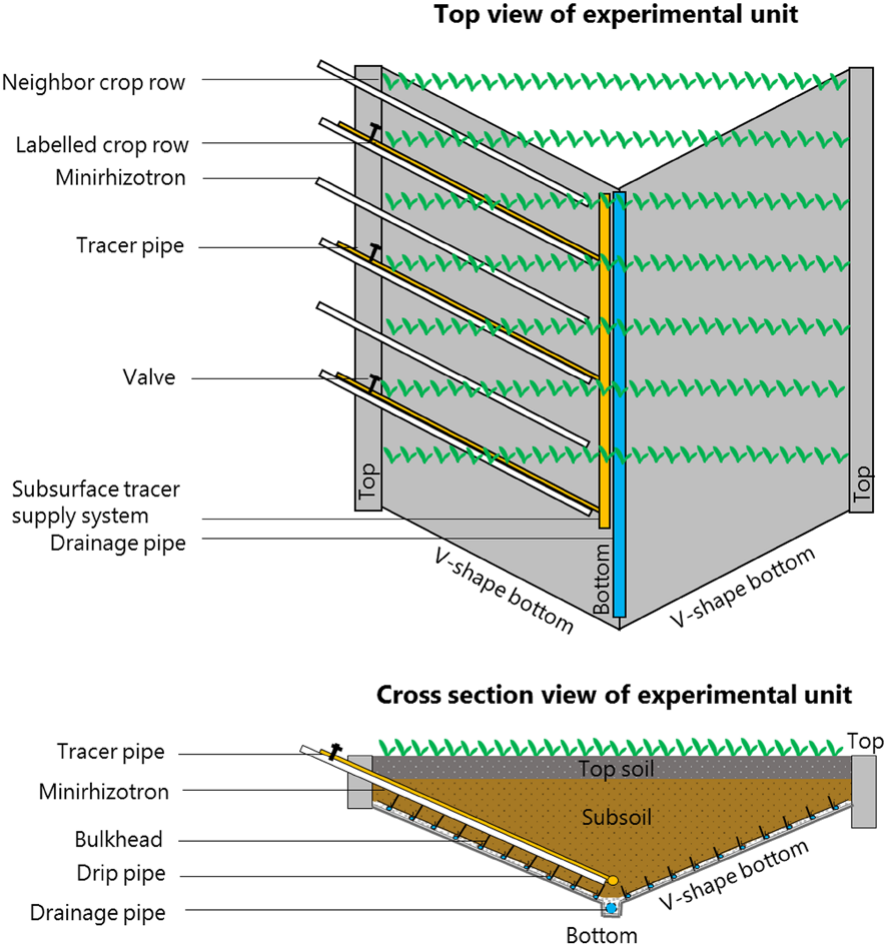
Schematic of the experimental set-up. Each unit of the RadiMax facility had a V-shaped bottom. Minirhizotrons were overlain on the bulkheads at 0.25 m intervals, sloping from the edge towards the middle of the facility. A subsurface tracer supply system involved 15 tracer pipes, set next to every second minirhizotron. The crop rows above the tracer pipes were labeled crop rows, and those above minirhizotrons were neighbor crop rows. A subsurface irrigation system was established along the bulkheads, enabling irrigation at ten soil depths separately

**Fig. 3:**
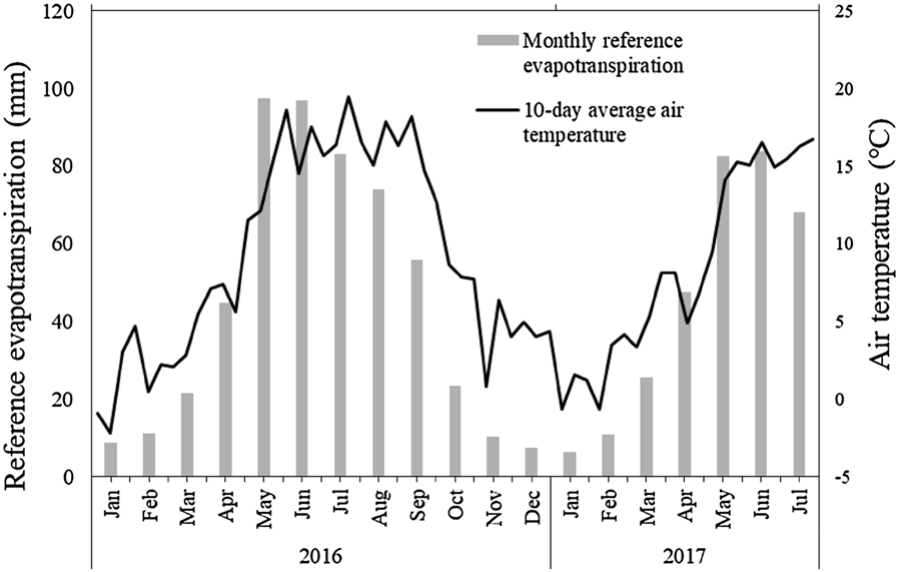
Meteorological conditions from January 1, 2016 to July 24, 2017. The figure shows monthly reference evaporation (mm) and 10-day average air temperature (°C)

**Fig. 4:**
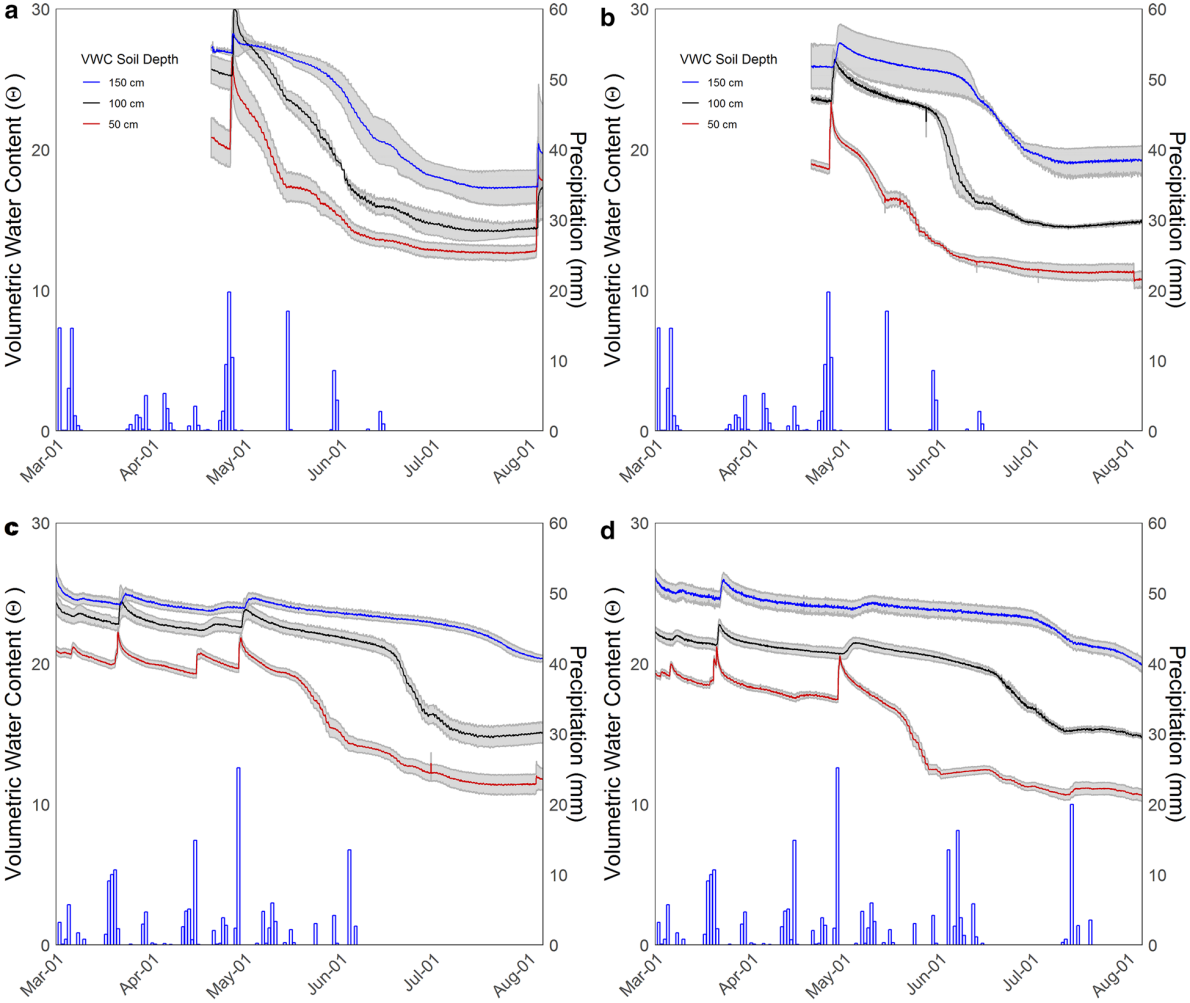
Daily precipitation and hourly volumetric water content (VWC) at 0.5 m, 1.0 m and 1.5 m soil depth. Four experiments were done in two seasons with rainout shelters used to create terminal drought stress in each experiment individually. Rainout shelters were used to cover winter wheat from 20 May to 24 May, from 27 May to 28 May and from 12 June to 21 July in Exp. 1 (**a**), and to cover spring barley from 15 June to 2 August in Exp. 2 (**b**) in 2016. In 2017, rainout shelters were used to cover spring barley from 7 June to 2 August in Exp. 3 (**c**) and ryegrass from 15 June to 15 July in Exp. 4 (**d**) in 2017

The RadiMax facility includes movable rain-out shelters to cover the plants and subject them to drought stress, as described by Svane et al. [32]. A subsurface irrigation system was established in the facility to create drought stress gradients along the crop rows by irrigating at increasing depths from the edge to the middle of the facility (Fig. 2). Weather data was obtained from a meteorological station located less than 700 m from the experimental site. The monthly reference evapotranspiration and 10-day average air temperature are shown in Fig. 3. The daily precipitation is shown in Fig. 4.

### Experimental set up

The facility consists of four units (Fig. 1), each 40 m long and 9.7 m wide. Each unit has a V-shaped bottom in the belowground. The vertical depth of the V-shaped bottom ranges from 1.1 to 3.0 m soil depth in units 1 and 2 and from 0.7 to 2.1 m soil depth in units 3 and 4. Ten bulkheads of 0.28 m height were inserted horizontally on each side slope of the V-shaped bottom. Drip pipes were placed along the bulkheads, which enables irrigation at 10 soil depths separately. The flux of each drip was 0.85 L h^−1^. The minirhizotrons (70 mm in outer diameter and 60 mm in inner diameter) were overlain on the bulkheads at 0.25 m intervals, sloping from the edge towards the middle of the facility [32]. In total, 15 tracer pipes from a subsurface tracer supply system were set next to every second minirhizotron at one end of each unit. A drainage pipe was located in the deepest part of the V-shaped bottom to drain excess soil water. The bottom of each unit was sealed by a plastic membrane. Within each unit, 150 crop rows planted at 0.25 m intervals can be grown above the minirhizotrons. Each experiment involved 30 crop rows, i.e., 15 neighbor crop rows and 15 labeled crop rows (Fig. 2), for further details, see [32].

### Semifield experiment

Each unit was filled with topsoil (0–0.4 m) and subsoil (below 0.4 m), which were taken from a local farm at 0–0.3 m soil depth and a field at 0.5–2.0 m soil depth, respectively. The soil is classified as a sandy loam. The soil texture, pH and soil fertility are shown in Table 2.

**Table 2:** Soil characteristics in the three RadiMax units

Unit	Soil depth (m)	Clay (< 2 ɥm) (%)	Silt (2–50 ɥm) (%)	Fine sand (50–500 ɥm) (%)	Coarse sand (500–2000 ɥm) (%)	Organic carbon (%)	Soil bulk density (g cm^−3^)	PH	P (mg kg^−1^)	K (mg kg^−1^)	Mg (mg kg^−1^)
1	0–0.4	0.12 ± 0.00	0.14 ± 0.03	0.45 ± 0.02	0.29 ± 0.01	0.015 ± 0.000	1.58 ± 0.04	7.10 ± 0.20	3.50 ± 0.00	10.50 ± 1.00	5.05 ± 0.30
	0.5–1.0	0.14 ± 0.02	0.14 ± 0.04	0.45 ± 0.05	0.26 ± 0.01	0.006 ± 0.004	1.76 ± 0.07	7.40 ± 0.20	0.90 ± 0.40	3.90 ± 0.00	3.15 ± 0.30
2	0–0.4	0.12 ± 0.01	0.13 ± 0.03	0.44 ± 0.01	0.29 ± 0.01	0.017 ± 0.004	1.59 ± 0.05	7.00 ± 0.40	4.00 ± 1.00	12.00 ± 4.00	5.20 ± 0.00
	0.5–1.0	0.14 ± 0.01	0.13 ± 0.01	0.47 ± 0.00	0.25 ± 0.02	0.006 ± 0.010	1.70 ± 0.06	7.50 ± 0.40	0.75 ± 0.70	3.55 ± 0.70	2.95 ± 0.70
3	0–0.4	0.12 ± 0.00	0.12 ± 0.01	0.46 ± 0.01	0.28 ± 0.01	0.018 ± 0.000	1.57 ± 0.01	6.85 ± 0.30	3.65 ± 0.30	11.00 ± 0.00	5.05 ± 0.10
	0.5–1.0	0.13 ± 0.00	0.13 ± 0.01	0.45 ± 0.00	0.28 ± 0.00	0.004 ± 0.003	1.70 ± 0.01	7.50 ± 0.20	0.80 ± 0.80	3.35 ± 0.30	2.90 ± 0.40

In all the units, the soil was plowed to a depth of 0.2 m and was harrowed before seeding. In Exp. 1, two winter wheat genotypes were sown in unit 1 on 1 October 2015. Crops were fertilized with 70 kg N ha^−1^ on 15 March and again on 11 May 2016. In Exp. 2, two spring barley genotypes were sown in unit 3 on April 12, 2016. N fertilizer was supplied at a rate of 70 kg ha^−1^ on the same day as sowing. In Exp. 3, seven spring barley genotypes were sown in unit 1 on 28 March and in unit 2 on April 4 in 2017. N fertilizer was added at the rate of 100 kg ha^−1^ on the sowing day. The sowing densities of wheat and barley were 350 seeds m^−2^ and 300 seeds m^−2^, respectively. In Exp. 4, four ryegrass genotypes were sown in unit 3 on September 27 in 2016. Fertilizers containing 40, 120, 50 and 94.5 kg N ha^−1^ were supplied on 27 September 2016, 16 March 2017, 16 May 2017 and 02 June 2017, respectively. The sowing density of ryegrass was 800 seeds m^−2^. Within each of the four experiments, the genotypes were sown in four replicates in a randomized plot design.

The subsurface irrigation system was manually controlled to irrigate at increasing depth along the V-shaped bottom. The soil moisture was measured by time domain transmission (TDT) sensors (Acclima, Inc., USA), which were installed at 0.5 m intervals along the V-shaped bottom with 0.25 m distance and in the west, middle and east of each unit at 0.5 m, 1 m and 1.5 m soil depths. The volumetric soil water content along the soil depth gradient is shown in Fig. 4. The irrigation and rain-out shelter were combined to create drought stress gradients along the crop rows. The subsurface water supply at increasing depths from the edge towards the middle of the facility makes it easy for plants growing towards the edge to reach the subsoil water, while plants along the planting rows towards the middle of the facility needed to grow deeper to access the water supply. The subsurface irrigation system was placed c. 0.28 m below the minirhizotrons. Weed and disease control were performed by herbicide and fungicide spraying as recommended according to local conditions.

### ^15^N tracer method

The ^15^N-labeled fertilizer was supplied to the labeled crop rows through the tracer supply system (Table 1). The ^15^N tracer was assumed to be evenly distributed along the labeled rows, following the depth gradient of the V-shaped bottom. The ^15^N uptake was measured by aboveground plant matter of the labeled rows (Fig. 2) and neighbor rows.

Spikes were sampled to measure ^15^N uptake at maturity in Exp. 1, Exp. 2, and Exp. 3 (Table 1). Samples were collected at four different intervals of each crop row on the labeled side of each unit (Table 3). The samples were oven-dried for 3 days at 75 °C. The dry matter was ground and placed into tin capsules for ^15^N analysis at the UC Davis Stable Isotope Facility, USA. The total N and ^15^N concentrations were obtained. The procedure for calculating of ^15^N enrichment was performed according to Walley et al. [35].

**Table 3:** Variations in grain N content and grain yield for different genotypes and species in three experiments

Exp	Species	Genotype	Grain yield (Mg DM ha^−1^)		Grain N content (kg ha^−1^)	
			0.87–1.74 m soil depth	1.74–2.61 m soil depth	0.87–1.74 m soil depth	1.74–2.61 m soil depth
1	Winter wheat	Hystar	9.3 a	7.7 a	95.7 a	81.2 a
		Tabasco	8.0 b	6.4 a	96.7 a	76.5 a

Exp	Species	Genotype	0.53–1.09 m soil depth	1.09–1.66 m soil depth	0.53–1.09 m soil depth	1.09–1.66 m soil depth
2	Spring barley	Evelina	4.6 a	3.8 a	54.2 a	44.6 b
		Laurikka	5.5 a	5.3 a	64.5 a	62.0 a

Exp	Species	Genotype	0.87–1.74 m soil depth	1.74–2.61 m soil depth	0.87–1.74 m soil depth	1.74–2.61 m soil depth
3	Spring barley	Laurikka	6.1 ab	6.5 a	73.9 a	81.3 ab
		Kenia	5.4 b	5.3 b	71.0 a	67.9 b
		Evergreen	6.0 ab	7.1 a	73.3 a	92.4 a
		Evelina	6.4 ab	6.7 a	89.9 a	93.0 a
		Invictus	7.0 a	7.5 a	83.6 a	96.4 a
		Prisma	6.1 ab	7.0 a	72.9 a	86.5 a
		Tocada	7.3 a	7.6 a	85.2 a	94.4 a

Grain N content and grain yield were measured for crops that grew at different soil depths. Different lowercase letters indicate significant genotypic differences at P < 0.05

### Minirhizotron method

Minirhizotrons provided an interface between a transparent tube and the soil, and a semiautomated minirhizotron camera was used to take images at intervals along the minirhizotrons, i.e., at increasing soil depths. Root images were recorded on 18 May in Exp. 1 and 3 June in Exp. 2 in 2016 and on 21 June in Exp. 3 and 23 May in Exp. 4 in 2017. Root depth was estimated based on these images, as the deepest soil depth where roots were visible on the images. Root intensity was measured by attaching transparent sheets with grid lines on the images and counting the number of roots crossing the grid lines [27].

### Statistical analysis

The estimated root depth, ^15^N uptake and ^15^N enrichment of different genotypes in the four experiments were analyzed using R statistical software [36]. A normal distribution test was performed with the Shapiro–Wilk method (P ≤ 0.05) prior to statistical testing for the equality of means by F-tests in analysis of variance (ANOVA). Tukey’s Honestly Significant Difference (HSD) (P ≤ 0.05) was used for post hoc analysis among genotypes. A linear mixed-effects model was used to take the block effects into account in Exp. 3 [37].

## Results

### Crop performance

The grain crops grew well in the facility and produced generally good yields, i.e., on average 7.9, 4.8 and 6.6 Mg DM of grain per hectare for winter wheat in Exp. 1 and spring barley in Exp. 2 and 3, respectively (Table 3). Genotype differences in yield were limited, but in Exp. 3, the old cultivar ‘Kenia’ showed a lower yield than the other genotypes. In Exp. 1 and 2 in 2016, there was a tendency towards higher yields and N uptake in the shallow part of the facility, with better water supply than the deep part. In Exp. 3 in 2017, the trend was opposite, with higher yields and N uptake in the deep part of the facility.

### Root growth and ^15^N uptake

Root depth development varied among the three species studied (Table 4), with ryegrass reaching 1.1 to 1.2 m depth, spring barley reaching 1.2 to 1.5 m and winter wheat reaching c. 1.65 m.

**Table 4:** ^15^N enrichment and root depth of different genotypes

Exp	Species	Genotype	Root depth (m)	^15^N enrichment (mg m^−1^)			
				0.98 m soil depth	1.50 m soil depth	2.02 m soil depth	2.54 m soil depth
1	Winter wheat	Hystar	1.64 a	2.84 a	2.24 a	0.12 a	0.08 a
		Tabasco	1.68 a	2.64 a	2.38 a	0.19 a	0.07 a

Exp	Species	Genotype	Root depth (m)	0.67 m soil depth	0.95 m soil depth	1.23 m soil depth	1.52 m soil depth
2	Spring barley	Evelina	1.23 a	2.59 a	2.47 a	2.07 a	1.73 a
		Laurikka	1.24 a	3.42 a	3.39 a	4.42 a	2.57 a

Exp	Species	Genotype	Root depth (m)	1.09 m soil depth	1.52 m soil depth	1.96 m soil depth	2.39 m soil depth
3	Spring barley	Laurikka	1.21 b	2.62 ab	0.55 a	0.09 ab	0.10 ab
		Kenia	1.26 ab	1.88 ab	0.36 a	0.09 b	0.08 b
		Evergreen	1.29 ab	1.23 b	0.55 a	0.11 a	0.10 a
		Evelina	1.32 ab	2.49 ab	0.58 a	0.10 ab	0.11 a
		Invictus	1.34 ab	1.87 ab	0.48 a	0.11 ab	0.10 a
		Prisma	1.42 ab	3.09 a	0.96 a	0.10 ab	0.10 a
		Tocada	1.48 a	2.05 ab	1.19 a	0.11 a	0.11 a

Exp	Species	Genotype	Root depth (m)	^15^N enrichment (atom% excess)			
				0.67 m soil depth	0.95 m soil depth	1.23 m soil depth	1.52 m soil depth
4	Ryegrass	Mercitwo 2 N	1.12 a	0.11 a	0.13 b	0.07 a	0.01 a
		Tetrastar 4 N	1.13 a	0.16 a	0.17 b	0.05 a	0.01 a
		Esquire 2 N	1.14 a	0.15 a	0.10 b	0.02 a	0.01 a
		Fabian 4 N	1.16 a	0.17 a	0.41 a	0.04 a	0.03 a

^15^N enrichment was measured at four soil depths from four intervals (i.e., 0.87–1.08 m, 1.39–1.61 m, 1.91–2.13 m, and 2.44–2.65 m in Exp. 1, 0.53–0.81 m, 0.81–1.09 m, 1.09–1.37 m, and 1.37–1.66 m in Exp. 2 and Exp. 4, and 0.87–1.30 m, 1.30–1.74 m, 1.74–2.17 m and 2.17–2.61 m in Exp. 3). Root images were taken by minirhizotron camera on 18 May in Exp. 1 and 3 June in Exp. 2 in 2016, and 21 June in Exp. 3 and 23 May in Exp. 4 in 2017. Root depth was defined as the deepest soil depth in these images where the roots were observed. Different lowercase letters indicated significant genotypic differences at P < 0.05

The results showed significant ^15^N uptake of wheat, barley and ryegrass in all four experiments, with a general decrease with soil depth. There was much higher ^15^N uptake from the two shallow soil depths in Exp. 1, 3 and 4 compared to the crop receiving ^15^N at larger depths. Within these three experiments, the maximum depth of ^15^N uptake corresponded to the measured rooting depth of the crop at the time of ^15^N injection. In the three experiments, limited uptake was estimated from the depth below the estimated average rooting depth, but no uptake was found from the deepest soil layer. In Exp. 2, there was significant ^15^N uptake at the four soil depths (Table 4), and the uptake depth thus clearly exceeded the rooting depth at the time of ^15^N injection.

In three of the experiments, there were no significant differences in root depth measured by the minirhizotrons among the genotypes, except in Exp. 3, where Tocada (1.48 m) was found to have significantly deeper roots than Laurikka (1.21 m) (Table 4).

**Fig. 5:**
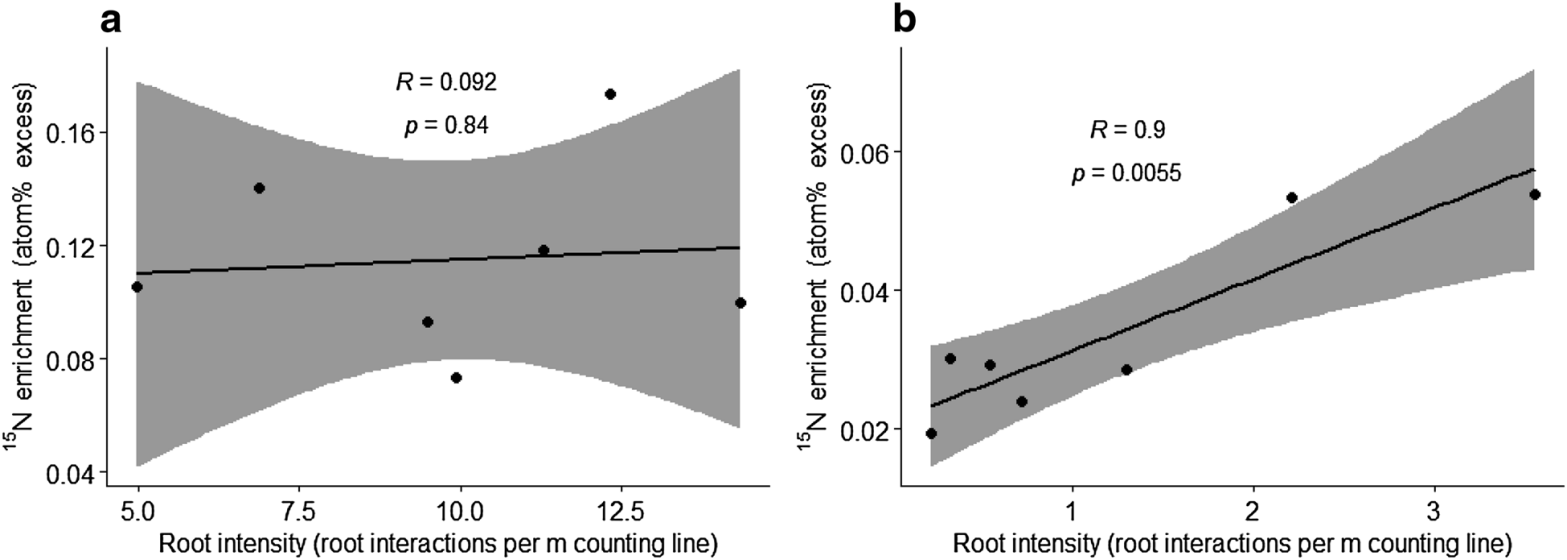
The correlations of deep root intensity and ^15^N enrichment in barley grain. The correlations were conducted on seven genotypes of barley at 0.87–1.30 m (**a**) and 1.30–1.74 m (**b**) soil depths in Exp. 3

When correlating ^15^N uptake with root intensity in Exp. 3, in the two upper layers 0.87–1.30 m and 1.30–1.74 m, a significant correlation was shown in the deeper layer (1.30–1.74 m; R = 0.90, P = 0.006), while no correlation was observed in the upper layer (0.87–1.30 m; R = 0.09, P = 0.844) (Fig. 5).

### ^15^N enrichment of labeled and neighbor rows

**Fig. 6:**
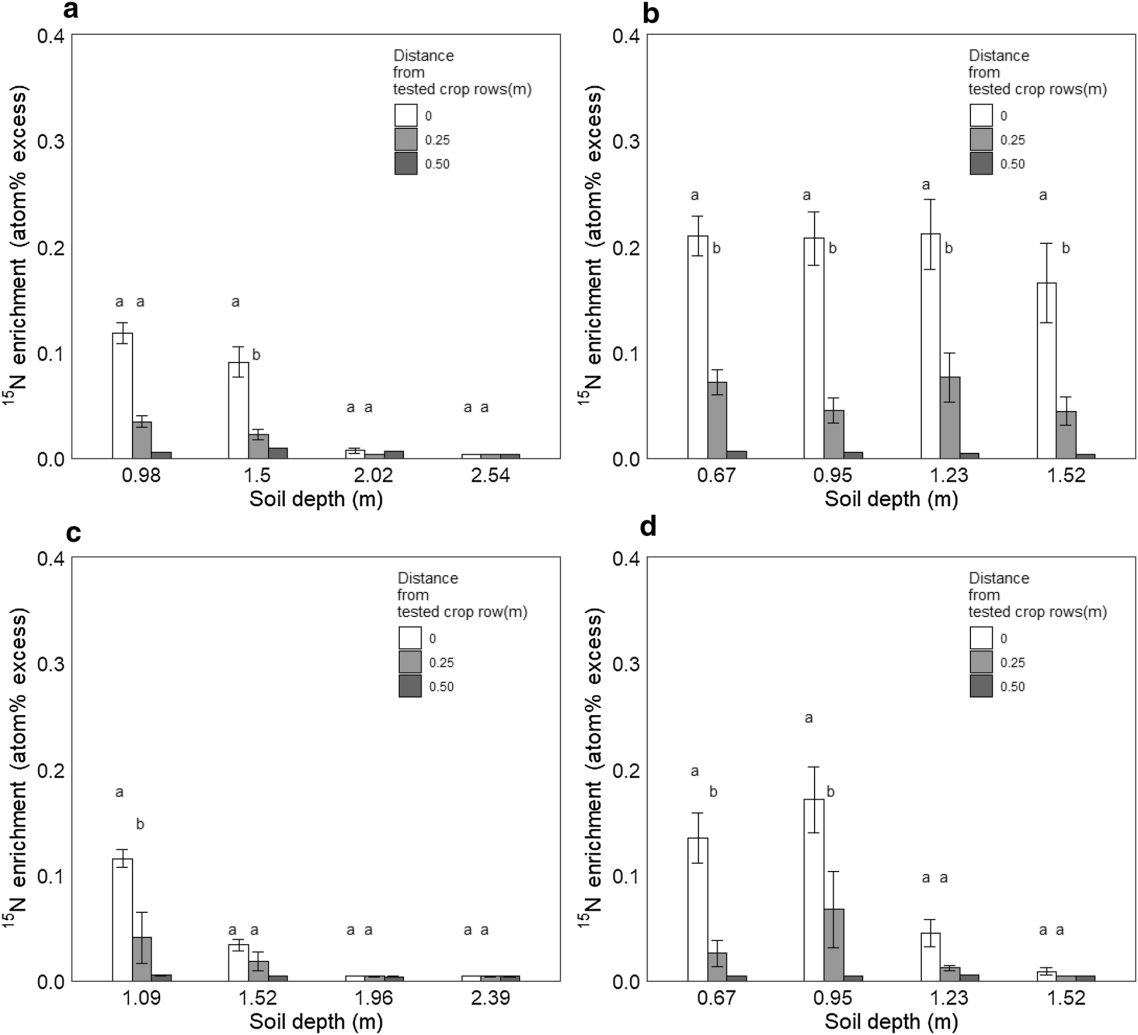
^15^N enrichment of the labeled and neighboring crop rows at four soil depths. The ^15^N enrichment measurements were performed on grain at harvest (**a** winter wheat in Exp. 1, **b** spring barley in Exp. 2, and **c** spring barley in Exp. 3) or on green tillers 25 days after labeling (**d** ryegrass in Exp. 4). The neighbor crop rows were 0.25 m or 0.50 m from the labeled crop rows. Different lowercase letters indicate significant differences among crop rows at P < 0.05. The furthest neighbor crop rows were only replicated once, and their statistical significance was ignored

To test the validity of the measurements in the facility we compared ^15^N uptake in the labeled crop lines to uptake in their neighbor rows, 0.25 m and 0.50 m away (Fig. 6). Significant uptake in neighbor rows may show either roots spreading across rows or movement of labeled N away from the injection point towards the neighbor row.

In all experiments, the ^15^N enrichment in the labeled row was higher than in neighbor rows 0.25 m away. The higher enrichment in the labeled row was observed from all injection depths where significant enrichment was observed, and the enrichment was generally 3 to 5 times higher in the labeled row than in the neighbor rows (Fig. 6). The effect was significant in most cases. In deep layers in Exp. 1, 3 and 4, where very little enrichment was observed, no difference between labeled and neighbor rows was observed. In Exp. 2, enrichment was observed at all four soil depths, and from all layers, the labeled genotypes showed significantly higher ^15^N enrichment than the neighbor rows (Fig. 6b). Very little ^15^N enrichment was observed in any of the samples collected 0.50 m away from the test rows in any of the experiments (Fig. 6).

## Discussion

### The subsurface tracer supply system

The subsurface tracer supply system is an innovative facility designed to apply tracers to the underground for deep root phenotyping. In this study, it enabled the supply of ^15^N-labeled fertilizer to the deep roots of wheat, barley and ryegrass along an increasing soil depth from 0.4 to 1.8 m or from 0.7 m to 2.8 belowground. By measuring aboveground ^15^N uptake at intervals along the depth gradient, depth activity was estimated. Additionally, the amount of ^15^N uptake can be related to the deep root density measured by the minirhizotrons. ^15^N as well as root growth and observation by the minirhizotrons is affected by a number of factors, such as heterogeneous soil compaction and moisture, and minirhizotron measurements rely on the appearance of visible roots on the tube surface and new image analysis strategies to separate new roots and dead roots or roots of other plants [38]. The semifield facility was designed to reduce these problems compared to direct field studies, where soil variability is often high. The ^15^N tracer method is able to detect the deep roots in situ. While the sloping subsurface tracer supply system is ideal to support studies of the tracer method in the RadiMax facility, a full-scale phenotyping approach would probably need to be based on a specific target injection depth, thereby labeling all rows within the unit could be conducted in future experiments.

### Relationship between ^15^N uptake and deep root growth of crop species

In this study, it was confirmed that ^15^N injection using driplines in the RadiMax facility and measuring its uptake could be used to study differences in deep rooting among crop species and to reveal differences among genotypes where expected differences are smaller. The ^15^N uptake of all crops was high when it was injected at shallow soil depths, where minirhizotrons showed significant root growth. For all crops, ^15^N enrichment was low from larger soil depths where observations did not show root growth at the time of ^15^N supply. A good agreement between the ^15^N uptake and root depth of crops under field conditions has previously been reported by Kristensen and Thorup-Kristensen [26]. ^15^N was taken up when placed within the soil layers where root growth was measured using the minirhizotron method, but no ^15^N uptake was found when the ^15^N was placed below the measured rooting depth [26].

### Relationship between ^15^N uptake and deep root growth of crop genotypes

In one of the four experiments, Exp. 3, where significant differences in deep rooting were observed among the genotypes, genotypic differences in ^15^N uptake and its relationship to root observations could be studied. Few significant genotype effects were found, but we did find a correlation between ^15^N tracer uptake and minirhizotron root observations in the deepest part of the root zone. For spring barley, significant differences were indicated in the shallowest soil depth interval studied, but as root density in that location was high for all genotypes, this may not be important for N uptake in this layer or indicates the ability of deep rooting. Other traits might be important for the effective utilization of soil N in this interval [25, 39]. Therefore, it is difficult to draw a conclusion on which method is superior because both of them had merits and limitations. Furthermore, the shallow and deep soil layers had different soil moisture contents, which can influence ^15^N uptake. In addition, ^15^N uptake also depends on the transpiration rate and N demand of the shoot [26]. Although a few significant genotypic differences were observed, and a good correlation between deep root length and deep ^15^N uptake in spring barley was observed, showing the potential of the method, it is also clear that the considerable variability of the measurements is a limitation of the method as it is. Reducing experimental variability will be important for the efficient use of the method for larger scale phenotyping.

Deep rooting traits are closely related to root uptake ability because deep-rooting genotypes can explore deep-stored resources and increase drought tolerance [6, 8, 40]. The deep rooting genotypes of spring barley generally had higher grain N content and yield than some of the shallow rooting genotypes in this study. Under terminal drought stress, water and nutrient use from subsoil are critical to reduce yield loss [41]. In this study, ‘Tocada’ with the deepest rooting had the highest ^15^N uptake at a larger soil depth.

A large number of studies have found that deep and branching root traits are cheap and effective way to improve root uptake ability and to reduce drought stress under water limited conditions [4, 6, 8, 42]. However, there is a lack of methods for phenotyping root traits and especially deep root traits in situ [21]. Therefore, many studies attempt to perform phenotyping for deep rooting on very young plants without well-developed root systems and grown in artificial growing media, which often fail to meet the expectations when grown in the field [18, 19, 43]. In contrast, the ^15^N tracer method is a direct way to phenotype deep rooting in situ. It is a further advantage that the tracer method measures the nitrogen uptake ability of deep roots, rather than just root growth, and potentially this can be applied to other tracers for water and other nutrients, using, e.g., ^2^H_2_O-labeled water or ^32^P-labeled fertilizer.

### ^15^N enrichment of neighbor crop rows

We hypothesized that roots of a crop row grow mainly below the row itself, and do not use resources from below neighbor rows. This was confirmed by the significantly higher ^15^N enrichment from the labeled crop rows than the neighbor rows at the root reached depth. Some ^15^N uptake did occur in the neighbor rows, but as the tracer itself will spread in the soil, to some degree towards the neighbor row, this indicates rather limited mingling with roots from neighbor lines, and that roots detected in a minirhizotron belong mainly to the row above it. The horizontal spread of roots can vary strongly among crops [21, 44, 45], but the horizontal spread of root systems of wheat, barley and ryegrass has rarely been measured due to technological bottlenecks. The ^15^N tracer method was useful for distinguishing the deep roots among crop rows in this study, and in previous studies it has even been used to map root distributions in intercropping systems [46].

## Conclusion

This study tested deep ^15^N placement through drip irrigation lines for deep root phenotyping of wheat, barley and ryegrass in four individual experiments in a new root phenotyping facility, RadiMax. The results showed significant ^15^N uptake of all crops from upper soil layers, but uptake from deeper layers was related to the rooting depth of the crops. Few significant genotypic variations in rooting depth and ^15^N uptake were detected, but a significant correlation between deep root length and deep ^15^N uptake was found among seven spring barley genotypes. The results show that the method has potential for use as a phenotyping method and is of interest because it measures actual root uptake activity rather than root growth, but they also indicate that improvements will be needed to reduce measurement variability. There were significant differences in ^15^N enrichment between the labeled crop rows and the neighbor rows, showing that roots grow mainly below the crop row itself, rather than mingling with roots of neighbor rows when the row spacing was 0.25 m.

## Data Availability

The datasets in this study are available from the corresponding author on reasonable request.

## Abbreviations

N: nitrogen
TDT: time domain transmission
